# A high-resolution melt curve toolkit to identify lineage-defining SARS-CoV-2 mutations

**DOI:** 10.1038/s41598-023-30754-1

**Published:** 2023-03-08

**Authors:** Alice J. Fraser, Caitlin Greenland-Bews, Daniel Kelly, Christopher T. Williams, LSTM Diagnostics Group, Daisy Bengey, Kate Buist, Karina Clerkin, Lorna S Finch, Susan Gould, Konstantina Kontogianni, Helen R Savage, Caitlin R Thompson, Jahanara Wardale, Rachel L Watkins, Dominic Wooding, CONDOR Steering Group, A. Joy Allen, Richard Body, Julian Braybrook, Peter Buckle, Eloïse Clark, Paul Dark, Kerrie Davis, Adam Gordon, Gail Hayward, Anna Halstead, Charlotte Harden, Colette Inkson, Naoko Jones, William Jones, Dan Lasserson, Joseph Lee, Clare Lendrem, Andrew Lewington, Mary Logan, Massimo Micocci, Brian Nicholson, Rafael Perera-Salazar, Graham Prestwich, Ashley Price, Charles Reynard, Beverley Riley, A. J. Simpson, Valerie Tate, Philip Turner, Mark Wilcox, Melody Zhifang, Richard Body, Emily R. Adams, Ana Cubas Atienzar, Thomas Edwards, David J. Allen

**Affiliations:** 1 London School of Hygiene and Tropical Medicine, London, UK; 2 Liverpool School of Tropical Medicine, Liverpool, UK; 3 Manchester University NHS Foundation Trust, Manchester, UK

**Keywords:** SARS-CoV-2, Infectious-disease diagnostics

## Abstract

The emergence of severe acute respiratory syndrome 2 (SARS-CoV-2) variants of concern (VOCs), with mutations linked to increased transmissibility, vaccine escape and virulence, has necessitated the widespread genomic surveillance of SARS-CoV-2. This has placed a strain on global sequencing capacity, especially in areas lacking the resources for large scale sequencing activities. Here we have developed three separate multiplex high-resolution melting assays to enable the identification of Alpha, Beta, Delta and Omicron VOCs. The assays were evaluated against whole genome sequencing on upper-respiratory swab samples collected during the Alpha, Delta and Omicron [BA.1] waves of the UK pandemic. The sensitivities of the eight individual primer sets were all 100%, and specificity ranged from 94.6 to 100%. The multiplex HRM assays have potential as a tool for high throughput surveillance of SARS-CoV-2 VOCs, particularly in areas with limited genomics facilities.

## Introduction

The emergence of severe acute respiratory syndrome 2 (SARS-CoV-2) variants poses a significant threat to ongoing efforts aimed at reducing the impact of the virus, both on individuals and health systems^1,2^. Variants containing mutations which are known or predicted to confer phenotypic changes are of particular interest from both a clinical and epidemiological perspective, as these may be associated with increased infectivity^3^, increased transmission^4^ or evasion of natural and/or vaccine-induced host immune responses^5,6^. Accumulation of advantageous mutations can lead to the emergence of a novel variant which has a competitive advantage, which can rapidly outcompete pre-existing variants within a population, as observed for the Delta^7^ and Omicron^8,9^ variants of concern (VOC).

Early detection and designation of variants containing advantageous mutations as either a variant of interest (VOI) or VOC is vital to inform public health responses. Where nations have been able to implement robust epidemiological surveillance systems based on next generation sequencing (NGS), it has been possible to track the emergence of VOCs in near real-time^10^. However, access to NGS is geographically inconsistent with many lower- and middle-income countries (LMICs) lacking access to the same infrastructure and resources, and consequently there is a surveillance gap in the molecular epidemiology of SARS-CoV-2 variants within many LMICs^11^.

The sequencing capacity within the African continent has increased dramatically during the pandemic, with over 100,000 SARS-CoV-2 genomes now sequenced within Africa and deposited at GISAID. This has been instrumental in uncovering the molecular epidemiology of the pandemic across Africa, and allowed the timely detection of the emergence of both Beta and Omicron VOCs. However, sequencing across the continent remains uneven, with the median positive samples sequenced ranging from 0.01% (Libya) to ~ 10% (The Gambia), and 16 countries still have no local sequencing capacity^11^. Accurate surveillance depends on the analysis of a large number of samples, collected at geographically and temporally spaced intervals, however the cost and infrastructure needed to implement the broad use of NGS across LMICs can be a barrier to its widespread use. Therefore, alternative methods of variant surveillance, which are cost-effective and easier to implement are needed to complement sequencing activities^12^.

To address this need, we have developed a high-resolution melt curve (HRM) assay for the detection of mutations, including single nucleotide polymorphisms (SNPs) and deletions, characteristic of SARS-CoV-2 variants. Primers were designed to give each amplicon a specific melting temperature to enable sensitive detection of each mutation. The use of HRM analysis has previously been applied to molecular diagnostics and surveillance in the context of infectious disease, including the detection of bacteria and antimicrobial resistance genes^13,14^, genotyping of *Plasmodium falciparum*^15^ and differentiation of viral genotypes by SNP analysis^16^.

In this report, we describe the design and evaluation of four separate multiplex HRM assays, to enable the identification of Alpha, Beta, Delta and Omicron VOCs by detection of the lineage defining mutations, of which E484K^2,17,18^ and 242–244 deletion^19^, have phenotypic implications. The collection of primer sets presented and the validated assays, whilst not a replacement for NGS, are intended for use as a molecular surveillance toolkit and offer an alternative method to enable efficient and cost-effective detection of SARS-CoV-2 variants and mutations.

## Materials and methods

### Viral RNA samples

Initial development and validation of the assays was performed using four in vitro cultured viruses maintained at LSHTM, for which stocks were obtained from the National Institute for Biological Standards and Controls (NIBSC): 19A SARS-CoV-2 (BetaCoV/Australia/VIC01/2020, CVA-GLA-1, CVA-GLA-2, CVA-GLA-3); two in vitro cultured viruses obtained from BEI resources: Beta VOC (hCoV-19/South Africa/KRISP-EC-K005321/2020), and Gamma VOC (hCoV-19/Japan/TY7-503/2021), and three cultured viruses maintained at LSTM: WT England B1 lineage (hCoV-19/England/20,092,096,704/2020), Alpha VOC (SARS-CoV-2/human/GBR/FASTER_372/2021) and Delta VOC (SARS-CoV-2/human/GBR/Liv_273/2021). In addition, two synthetic SARS-CoV-2 RNA controls (Twist Bioscience, USA) were used for early assay development, which were representative of Alpha VOC (England/205,041,766/2020) and Beta VOC (South Africa/KRISP-EC-K005299/2020).

### Clinical samples

A field evaluation to determine assay performance was undertaken using clinical samples collected from participants as part of the Facilitating Accelerated Clinical Validation Of Novel Diagnostics for COVID-19 (FALCON) study at the Liverpool John Lennon airport testing site. Subjects who required COVID-19 RT-PCR testing and who presented to a drive-through regional testing centre in the community were prospectively invited to participate. Informed consent was obtained from all subjects and/or their legal guardian(s). If subjects provided verbal consent, specimens were collected using combined oral and nasopharyngeal swabs (COPAN UTM-RT Diagnostics, Italy) and stored in 1 mL of universal transport media (UTM). Ethical approval was obtained from the National Research Ethics Service and the Health Research Authority (IRAS ID:28,422, clinical trial ID: NCT04408170). All methods were performed in accordance with the relevant guidelines and regulations. Patient specimens were collected using either combined throat and nasal swabs or nasopharyngeal swabs (COPAN UTM-RT Diagnostics, Italy) and stored in 1 mL of universal transport media (UTM).

Samples collected in the FALCON study were confirmed SARS-CoV-2 RNA-positive using the TaqPath™ COVID-19 CE-IVD RT-PCR Kit (ThermoFisher Scientific, USA). Based on epidemiological data were defined as either (i) presumed Alpha (N = 30) if collected between 15th and 22nd January 2021 when Alpha was the most commonly circulating variant in the UK, or (ii) presumed Delta (N = 30) if collected between 14th and 25th June 2021 when Delta was the most commonly circulating variant in the UK. SARS-CoV-2 RNA-negative samples from the FALCON study were used for specificity testing (N = 19). A further 16 samples collected between 13th and 21st December 2021 when Omicron was the most commonly circulating variant in the UK were used in evaluation of the Omicron assay.

### RNA extraction

RNA was extracted from in vitro cultured virus and clinical specimens in UTM transport media using the QIAamp Viral RNA Kit (QIAGEN, Germany), following the manufacturer’s protocol, and implemented as either a manual workflow for the viral cultures, or as an automated workflow using the QIAcube HT platform (QIAGEN) for clinical samples. Purified RNA was eluted in 50 μl of elution buffer stored at -20 °C until use.

### cDNA synthesis

Extracted RNA was diluted 1:1000 in molecular grade water (Thermo Fisher Scientific, USA) before cDNA generation by random-primed reverse transcription using the SuperScript IV First Strand Synthesis System (Invitrogen, USA). The thermal profile was modified from the manufacturer’s instructions as per the ARTIC PCR protocol; the reverse transcription was carried out at 42 °C for 50 min, followed by 70 °C at 10 min to inactivate the RT enzyme.

### Sequencing

Sequencing of SARS-CoV-2 genomes was performed using the ARTIC SARS-CoV-2 sequencing protocol^20^ on the Oxford Nanopore Technology (UK) MinION device. The ARTIC V3 primer sets were purchased as pools (Integrated DNA Technologies, USA), and the PCR and library preparation were carried out according to the ARTIC V3 sequencing protocol^20^. For suspected Omicron samples (based on collection date), the updated ARTIC V4.1 primer sets were utilised (Integrated DNA Technologies, USA). All PCR assays used the Q5® Hot Start High-Fidelity 2X Master Mix (New England Biolabs, USA), 10 μM primer pools, and a thermal cycling profile of a 30 s 98 °C heat inactivation, followed by 25 cycles of a 15 s denaturation at 98 °C and a five-minute annealing/extension at 65 °C. Library preparation was carried out using the Ligation Sequencing Kit (SQK-LSK109) and Native Barcoding Expansion Kits (EXP-NBD104 and EXP-NBD114; all Oxford Nanopore Technologies, UK). Enzymes for barcode and adapter ligation were purchased from New England Biolabs (USA), and AMPure XP beads (Fisher Scientific, USA) were used during the library preparation. Sequencing was carried out using an R.9.4.1 flow cell on a MinION device.

### Bioinformatics analyses

Basecalling was done via MiniKnow (v4.2.8), with demultiplexing and read filtering using Guppy (v5.0.7.). The ARTIC pipeline^20^ was then used to assemble a consensus genome, BAM files, and variant calling file with *–normalise 200 –threads 4.* The pipeline performs a reference alignment of basecalled reads using minimap and aligns the consensus sequence against the reference using Muscle. Automated rapid variant calling was carried out using EPI2ME Desktop Agent v3.3.0 with the ARTIC + NextStrain analysis pipeline, and via the vcf file generated via ARTIC. Genomes were analysed in Tablet (v1.21.02.08)^21^ for manual inspection of genomes and coverage. All sequence data is deposited in BioProject PRJNA936677 [https://www.ncbi.nlm.nih.gov/bioproject/PRJNA936677].

### HRM primer design

A total of 799 complete SARS-CoV-2 genomes from VOC lineages Alpha, Beta, Delta, Gamma and Kappa, and genomes sequenced from samples collected in England prior to the emergence of the Alpha VOC, were downloaded from GISAID (https://gisaid.org/). These were aligned using MAFFT version 7.453 with the –auto and –nuc parameters enabled^22^ alongside the GISAID reference sequence (hCoV-19/Wuhan/WIV04/2019) and all other sequences derived from RNA samples used in initial development. Primer pairs were designed to target seven different SARS-CoV-2 VOC or VUI lineage-defining mutations (Table 1):

- Orf1ab gene, deletion positions 3675–3677 [Orf1ab_del.3675–3677]
- Spike gene, deletion positions 156–157 [S_del.156–157]
- Spike gene, deletion positions 242–244 [S_del.242–244]
- Spike gene, substitution position 417 [S_K417N]
- Spike gene, substitution position 484 [S_E484K]
- Spike gene, substitution position 681 [S_P681H/R]
- Nucleoprotein gene, substitution position 3 [N_D3L]

**Table 1:** Description of the SARS-CoV-2 genome targets for, and detail of the sequences of, the seven primer sets designed in this study to amplify variant-defining mutations, and that were subsequently incorporated into multiplex assays A-D. Orf1ab = Open reading frame 1ab, S = Spike, N = Nucleocapsid.

Gene	Mutation type targeted	Amino acid position targeted	Primer set name	Variant						Forward primer sequence (5′–3′)	Reverse primer sequence (5′–3′)
				19A	Alpha	Beta	Gamma	Delta	Omicron		
Orf1ab	Deletion	3675–3677	Orf1ab_del.3675–3677		X	X	X		X	TGACATGGTTGGATATGGTT	ACAGCTGATGCATACATAACACA
S	Deletion	156–157	S_del.156–157					X		ACCACAAAAACAACAAAAGTTGG	TTCGCACTAGAATAAACTCTGAACTC
S	Insertion	214	S_EPE						X	CCTATTATAGTGCGTGAGCCA	CTATGTAAAGCAAGTAAAGTTTGAAACCT
S	Deletion	242–244	S_del.242–244			X				CAGGGTTTTTCGGCTTTAGA	AAGAATCACCAGGAGTCAAA
S	Substitution	417	S_K417N			X	X		X	GAAGTCAGACAAATCGCTCCA	AACGCAGCCTGTAAAATCATC
S	Substitution	484	S_E484K			X	X			CTATCAGGCCGGTAGCACAC	AAAGGAAAGTAACAATTAAAACCT
S	Substitution	681 (P > H)	S_P681H/R		X				X	TGACATACCCATTGGTGCAG	TAGTGTAGGCAATGATGGATTGA
S	Substitution	681 (P > R)	S_P681H/R					X			
N	Substitution	2	N_D3L		X					AACAAACAAACTAAAATGTCTGATA	TTACTGCCAGTTGAATCTGA

Primer sets were designed either to target conserved sites flanking mutations of interest, or to directly bind the mutation with the 3’ base of the primer. In case of the former, a shift in melt temperature (Tm) is detected if the mutation is present, whereas in the latter, the presence of the mutation prevents primer binding and ablated amplification. Primers were designed using Primer3Plus (https://www.bioinformatics.nl/cgi-bin/primer3plus/primer3plus.cgi), OligoCalc (http://biotools.nubic.northwestern.edu/OligoCalc.html) nearest neighbour method was used to estimate amplicons’ Tm^23^, all primer pairs were analysed for specificity using Primer-BLAST^24^. Final primer sequences are detailed in Table 1.

### HRM assays

Initially, three multiplex HRM assays, each containing three different primer pairs were developed and evaluated in this study. These assays identified combinations of variant-defining mutations for SARS-CoV-2 VOCs, with a focus on differentiation of Alpha from 19A, and later Delta from Alpha, to understand the epidemiology of these variants at the time in the UK.

Each assay was performed using 2.5 μL of RNA template and in 12.5 μl final reaction volumes, using the SuperScript™ III One-Step RT-PCR System with Platinum™ Taq DNA Polymerase kit (ThermoFisher, USA), with final reagent quantities as follows: 1X reaction mix, 0.25 μL of SuperScript™ III RT/Platinum™ Taq Mix, 1X EvaGreen® dye (Biotium, USA), and primers added to their optimised concentration (Table 2).

**Table 2:** Optimised final reaction primer concentrations for primer set in each multiplex assay, A-D. Primer set names as described in Table 1.

Primer set	Final forward primer concentration (nM)	Final reverse primer concentration (nM)
Multiplex A		
S_del.156–157	100	150
S_K417N	150	225
N_D3L	600	900
Multiplex B		
S_E484K	300	450
S_del.242–244	150	225
S_P681H/R	150	225
Multiplex C		
Orf1ab_del.3675–3677	200	300
S_del.242–244	200	300
N_D3L	175	262.5
Multiplex D		
S_del.156–157	100	100
S_K417N	150	150
N_D3L	600	600
S_EPE	250	250

Reactions were performed using the RGQ 6000 5-plex HRM platform (Qiagen, Germany) with the following thermal cycle profile: reverse transcription at 50 °C for 15 min, initial denaturation at 95 °C for 5 min, followed by 40 cycles of 95 °C for 10 s, 56 °C for 30 s and 72 °C for 20 s. HRM was then performed, melting from 73 °C to 85 °C, acquiring data to the HRM channel in 0.1 °C increments, with a 2 s stabilisation between each step. For each assay a reference control of 19A strain SARS-CoV-2 RNA, confirmed by WGS to be negative for all mutations of interest, and a no template negative control were included.

### Analysis of HRM assay data

Primary data were analysed using the RGQ system software (v2.3.5, Build 1, QIAGEN) and additional data analyses were performed in Prism (v9, GraphPad, USA).

**Figure 1:**
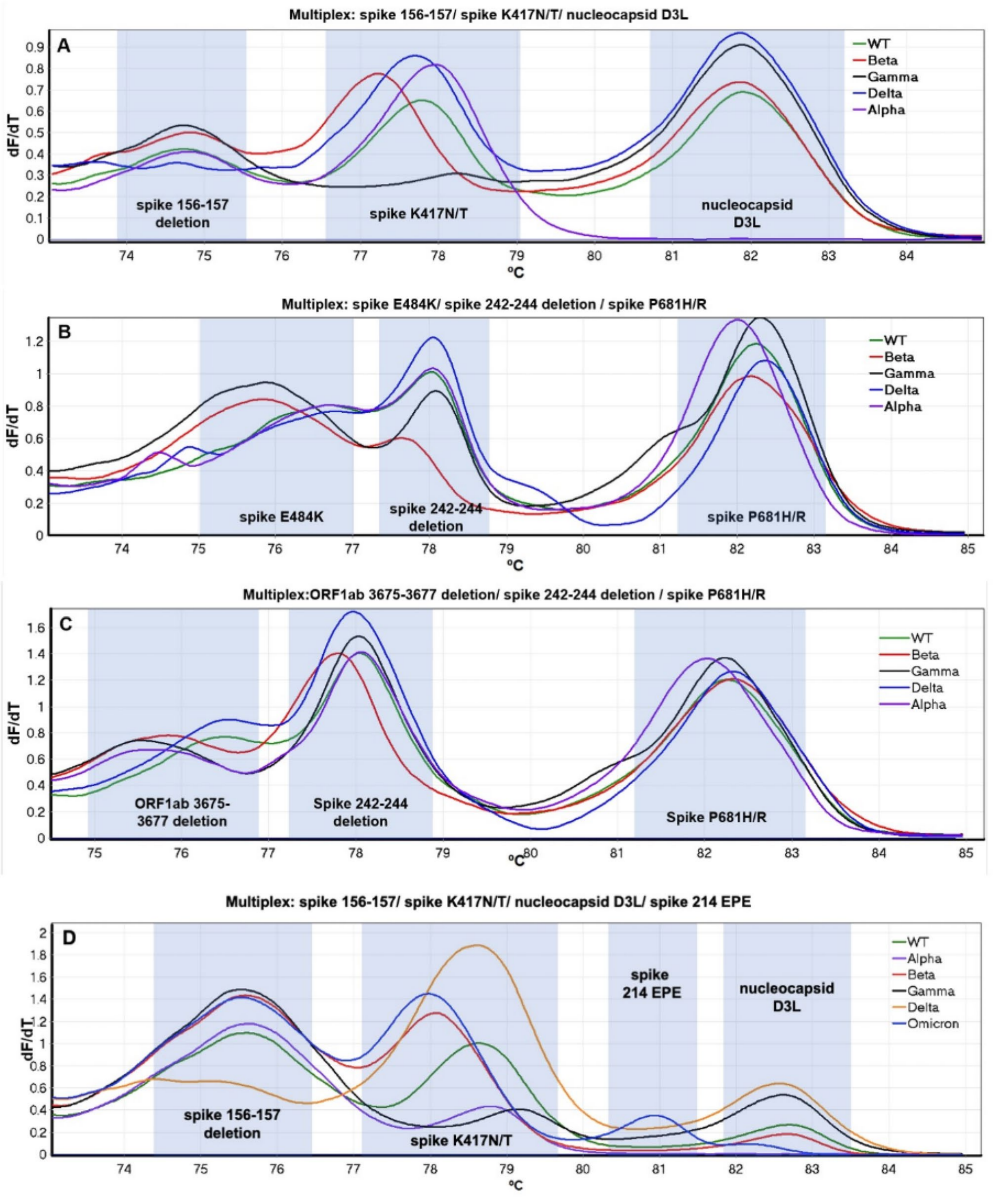
High resolution melt profiles (derivative, dF/dT) of multiplex assays A–D: (**A**) Multiplex A, targeting S_del.156–157, S_K417N and N_D3L, (**B**) Multiplex B, targeting S_E484K, S_del.242–244 and S_P681H/R, (**C**) Multiplex C, targeting Orf1ab_del.3675–3677, S_del.242–244 and S_P681H/R, and (**D**) Multiplex D, targeting S_del.156–157, S_K417N, N_D3L and S_EPE (all mutations and primer sets as described in Table 1). Data shows evaluation in vitro cultured SARS-CoV-2 strains, where the variant type had been determined by WGS, as follows: 19A (green line), Alpha VOC (purple line) SARS-CoV-2/human/GBR/FASTER_372/2021, Beta VOC (red line) hCoV-19/South Africa/KRISP-EC-K005321/2020, Gamma VOC (black line) hCoV-19/Japan/TY7-503/2021, Delta VOC (blue line) SARS-CoV-2/human/GBR/Liv_273/2021 and Omicron VOC RNA derived from a clinical specimen where SARS-CoV-2 RNA was detected at Ct < 30 and with known complete genome sequence.

Data was visualised as negative first derivative plots (Fig. 1), and Tm values were recorded for each peak. Thresholds for analysis were determined empirically during initial optimisation experiments to be as follows:

- Orf1ab_del.3675–36,770.40 dF/dT
- S_del.156–1570.35 dF/dT
- S_del.242–2440.20 dF/dT
- S_K417N0.20 dF/dT
- S_E484K 0.43 dF/dT
- S_P681H/R0.20 dF/dT
- N_D3L0.20 dF/dT

For primer sets targeting the S_del.156–157 and S_E484K, ORF1ab_del.3675–3677, primer-dimer curves present below 75 °C were excluded.

**Figure 2:**
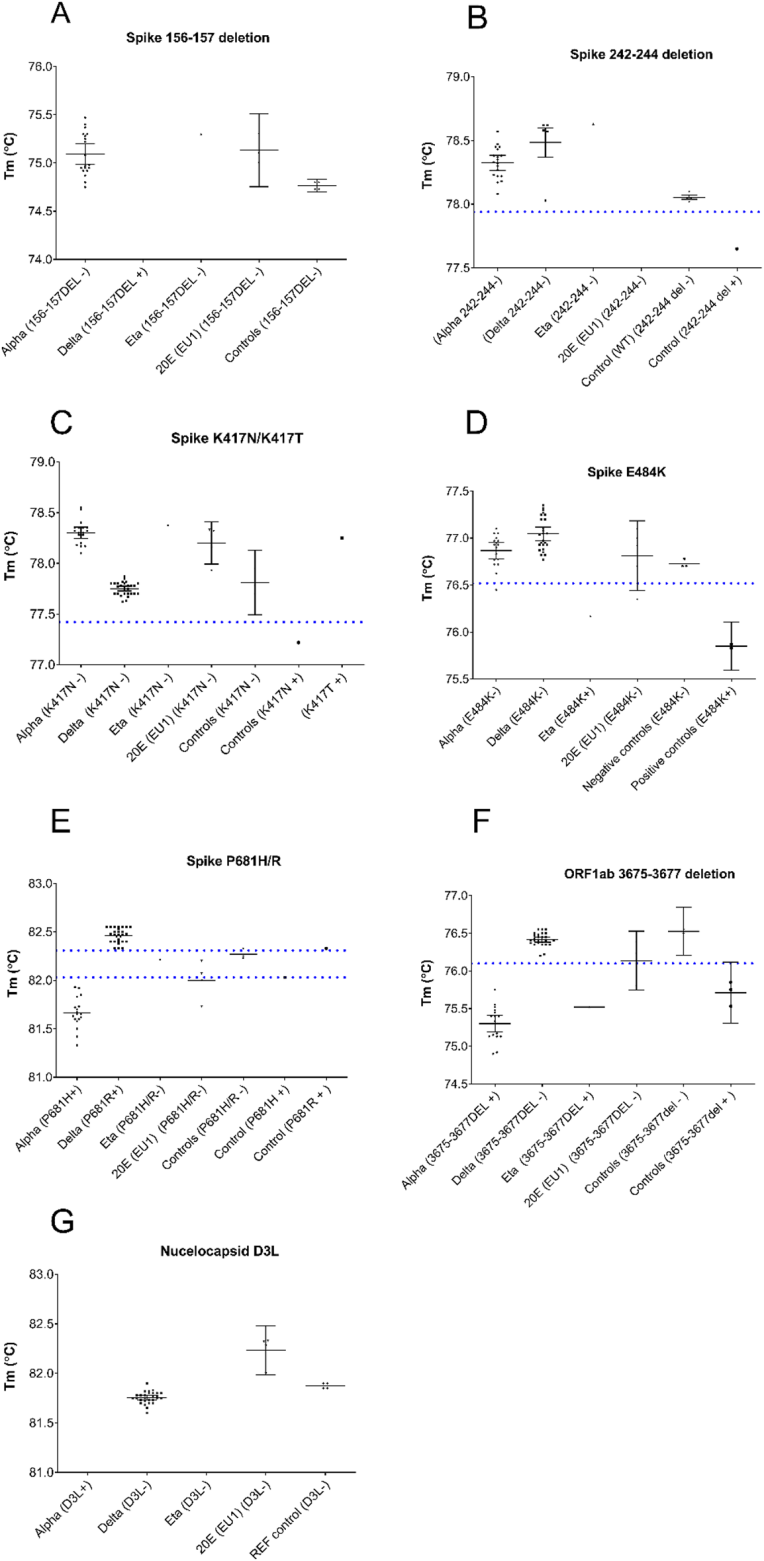
Melt temperature (Tm, °C) value distributions (with 95% confidence intervals) for clinical samples collected 15th–22nd January 2021 (Alpha, N = 30) or collected between 14 th and 25th June 2021 (Delta, N = 30) where SARS-CoV-2 RNA was detected at Ct < 30 and with known complete genome sequences. Data are shown for each primer set (as described in Table 1): (**A**) S_del.156–157, (**B**) S_del.242–244, (**C**) S_K417N, (**D**) S_E484K, (**E**) S_P681H/R (**F**) Orf1ab_del.3675–3677, and (**G**) N_D3L. Blue dotted lines represent the positivity thresholds (shown in Table 3), which when crossed indicates the presence of the mutation. Positivity thresholds were calculated by the addition or subtraction of two standard deviations surrounding the mean Tm, of samples confirmed negative for the mutation.

Assay interpretation details are shown in Table 3. The Tm threshold for calling a result based on a Tm shift was set by calculating the average Tm for all samples confirmed negative for the mutation and then adding two standard deviations in the direction of the temperature shift (Fig. 2).

**Table 3:** Validated melt temperature (Tm) values (°C) for peak Tm used in interpretation of high-resolution melt data that indicate sequences positive or negative for the targeted mutation, and threshold boundaries for determining positivity, by primer set (as described in Table 1). * In Omicron VOC, del3674-3676.

Assay	Mutation	Associated variants	Peak Tm (°C) indicating absence of mutation	Peak Tm (°C) indicating presence of mutation	Boundaries for determining presence of mutation (°C)	VOCs identified
A	S_del.156-157	Delta	75.06	No peak	No peak	Alpha, Delta
	S_K417N	Beta, Gamma, Omicron [BA.1]	77.99	77.22	<77.42	
	N_D3L	Alpha	81.76	No peak	No peak	
B	S_E484K	Gamma	76.94	75.96	< 76.52	Alpha, Beta, Gamma, Delta, Omicron [BA.1]
	S_E484A	Omicron				
	S_del.242-244	Beta	78.33	77.65	< 77.94	
	S_P681H	Alpha, Omicron [BA.1]	82.12	81.68	<82.03	
	S_P681R	Delta	82.12	82.46	>82.31	
C	Orf1ab_del.3675-3677	Alpha, Beta, Gamma, Omicron [BA.1]*	76.39	75.37	< 76.10	Beta, Delta
	S_del.242-244	Beta	78.33	77.65	< 77.94	
	S_P681H	Alpha, Omicron [BA.1]	82.12	81.68	<82.03	
	S_P681R	Delta	82.12	82.46	>82.31	
D	S_del.156-157	Delta	75.06	No peak	No peak	Alpha, Delta, Omicron [BA.1]
	S_K417N	Beta, Gamma, Omicron [BA.1]	77.99	77.22	<77.42	
	N_D3L	Alpha	81.76	No peak	No peak	
	S_EPE	Omicron	No peak	80.93	Presence of peak

### Rapid development of primers for the emerging Omicron BA.1 variant

In response to the emergence of the Omicron BA.1 VOC during the HRM assay development, a primer set was designed targeting the lineage-defining insertion of three amino-acid residues, glutamate-proline-glutamate (EPE) at position 214 in the spike protein in Omicron BA.1 lineage^14^. Primers were designed so the forward primer sits on the insertion and amplifies when the mutation is present. The Omicron primers were first optimised as a singleplex assay and evaluated using 16 samples confirmed as Omicron BA.1 by sequencing, alongside ten Alpha and ten Delta SARS-CoV-2 samples. The primers were designed to be compatible with Multiplex A, and their compatibility was demonstrated using single RNA samples of each VOC.The target of the Omicron primer set, ins214EPE, is only present in the Omicron BA.1 lineage, and further primer sets would be required in order to detect other Omicron lineages. This is potentially facilitated by the presence of unique mutations in all Omicron sublineages so far encountered^25^. This is highlighted to illustrate the speed at which this assay can be updated to accommodate emerging VOCs.

## Results

Initially, each primer pair (Table 1) targeting a specific mutation of interest was assayed individually against a panel of SARS-CoV-2 strains, including 19A, and VOCs Alpha, Beta, Gamma and Delta to define the melt curve profile and peak melting temperature (or absence thereof for primers designed for target drop-out) (Table 3).

Subsequently, the individual primer pairs were iteratively evaluated in multiplex formats to develop combinations that would result in HRM profiles that were variant-defining, by simultaneously targeting mutations that differentiated between VOCs . Ultimately, three multiplex assays, A-C, each targeting three mutations, were validated that could differentiate VOCs from the 19A strain prototype, and further, could discriminate between at least two different VOCs (Fig. 1 and Table 3).

To understand the performance of these assays in variant-calling of VOCs from clinical samples, a field evaluation was undertaken using archived clinical specimens collected at a COVID testing site in North West England (Fig. 2). In parallel to analysis by HRM, the complete SARS-CoV-2 genome sequence from each specimen was determined to confirm the VOC present and allow sensitivity and specificity of the HRM assay to be calculated.

### Sequencing of clinical samples

Of the 30 samples collected in January 2021 21 (70.0%) were confirmed as Alpha VOC by NGS and 29/30 (96.7%) samples collected in June 2021 (96.7%) were confirmed as Delta VOC by NGS.

### Sensitivity and specificity of the assays

Sensitivity and specificity of each primer pair to identify mutations was determined using NGS data. All primer sets had a sensitivity of 100%, with specificity values ranging from 96.00% to 100% (Table 4). The overall agreement between the HRM assays and sequencing was 98.10% (Cohen’s Kappa 0.962).

**Table 4:** **(A)** Sensitivity and specificity percentages (including 95% confidence intervals, 95% CI) for all primer sets (as described in Table 1), using data generated during validation with clinical samples collected 15th–22nd January 2021 (Alpha, N = 30) or collected between 14th and 25th June 2021 (Delta, N = 30) where SARS-CoV-2 RNA was detected at Ct < 30 and with known complete genome sequences. **(B)** Sensitivity and specificity percentages (including 95% confidence intervals, 95% CI) for multiplex assay A, targeting S_del.156–157, S_K417N and N_D3L (mutations and primer sets as described in Table 1). Sensitivity and specificity are based on the ability of multiplex A assay to discriminate between the two VOCs Alpha and Delta in clinical samples collected 15th–22nd January 2021 (Alpha, N = 30) or collected between 14th-25th June 2021 (Delta, N = 30) where SARS-CoV-2 RNA was detected at Ct < 30 and with known complete genome sequences.

**(A)**				
	**Sensitivity**	**(95% CI)**	**Specificity**	**(95% CI)**
Orf1ab_del.3675–3677	100	(84.56–100)	97.06	(84.67–99.93)
S_del.156–157	100	(87.66–100)	100	(87.23–100)
S_del.242–244	100	(2.50–100)	100	(88.78–100)
S_K417N	100	(2.50–100)	98.25	(90.61–99.96)
S_E484K	100	(29.24–100)	96	(86.29–99.51)
S_P681H	100	(82.35–100)	94.59	(81.81–99.34)
S_P681R	100	(88.06–100)	96.30	(81.03–99.91)
N_D3L	100	(78.20–100)	97.3	(85.84–99.93)

**(B)**				
Multiplex A	**Detection of Alpha VOC**			
	**Sensitivity (%)**		**Specificity (%)**	
	100 (80.46–100)		97.14 (86.19–99.93)	
	**Detection of Delta VOC**			
	**Sensitivity (%)**		**Specificity (%)**	
	100 (88.06–100)		100 (86.77–100)	

The three individual primer pairs with the highest sensitivity and specificity were those combined as Multiplex A (targeting S_del.156–157 / S_K417N / N_D3L). Evaluating the performance of Multiplex A, this assay was found to be 100% sensitive for the detection both Alpha (95% CI: 80.49–100) and Delta (95% CI: 88.06–100). Specificity was 97.14% (95% CI: 86.19–99.93) for Alpha detection and 100% (95% CI: 86.77–100) for Delta detection.

Notably, five samples collected in January 2021 (and so epidemiologically presumed as Alpha VOC) displayed HRM profiles for S_del.156–157 and S_K417N, but produced HRM curves in the N_D3L assay, which should result in target drop-out with the Alpha VOC. Analysis of sequence data for these five samples confirmed that all five were a different variant, 20E(EU1), explaining the discrepant result.

### Omicron assay

During the period of development of this assay, a new VOC, Omicron, emerged and we used the early sequence data available at the time through GISAID to modify Multiplex A to accommodate primers for detection of the emerging Omicron BA.1 VOC (Table 1), and defined as Multiplex D (Table 3).

**Figure 3:**
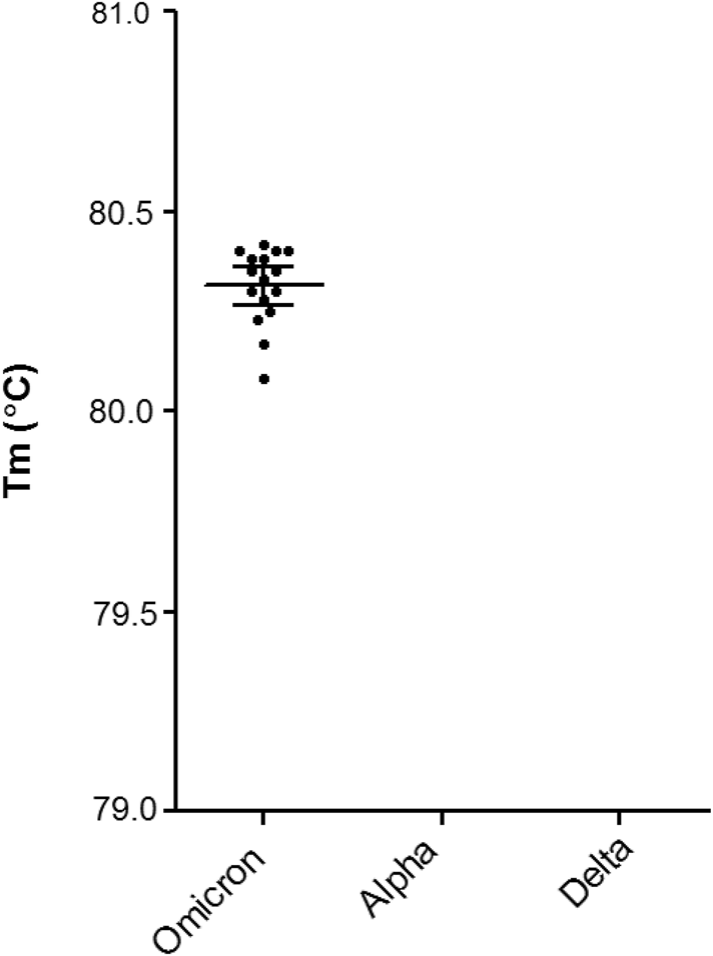
Melt temperature (Tm, °C) value distributions (with 95% confidence intervals) for clinical samples collected 15th-22nd January 2021 (Alpha, N = 10) or collected between 14th and 25th June 2021 (Delta, N = 10) or collected between December 2021–January 2022 (Omicron, N = 16) where SARS-CoV-2 RNA was detected at Ct < 30 and with known complete genome sequences.

The Omicron BA.1 primer set produced a distinct Tm peak at 80.3 °C. The primers were able to detect 16/16 Omicron positive samples (Sensitivity 100%, 95% CI: 79.4–100%), and did not produce a peak for 20 non-Omicron SARS-CoV-2 (10 Delta, 10 Alpha) positive samples (Fig. 3).

## Discussion

Here we describe a collection of HRM assays for the detection of mutations associated with major SARS-CoV-2 VOCs identified as of March 2022. Adopting a toolbox approach, we present primer sets for individual mutations of epidemiological importance that can be combined in multiplex formats, as well as examples of multiplex reactions that generate HRM profiles which distinguish between VOCs.

Alternative approaches to NGS for surveillance of VOCs and/or epidemiologically important mutations is a rapidly expanding area. Commercially available molecular assays for SARS-CoV-2 VOCs have been developed, including the TIB MolBio ViRSNiP^26^ tests, however these tests only identify a single SNP or deletion per assay, potentially requiring a panel of tests for comprehensive surveillance. Elsewhere, academic groups have developed assays for particular epidemiologically important mutations using either hydrolysis probes^27^, molecular beacons^28^ or isothermal methods^29^. However, the HRM approach we present is the first development of a comprehensive tool kit of primers that can be combined in response to local epidemiology, and furthermore is readily adaptable to the emergence of new VOCs, as demonstrated by the incorporation of primers for detection of the Omicron BA.1 VOC in our assay.

Given their demonstrated adaptability and ease of implementation, these HRM assays have the potential to improve access to testing and surveillance for SARS-CoV-2 VOCs in areas with either limited or exhausted sequencing capacity, which will be of particular benefit in LMIC settings. Compared to whole genome sequencing, the HRM assay has a faster setup and run time, and a more streamlined subsequent data analysis, to enable more rapid, and higher throughput, variant detection in comparison with NGS approaches. This would in turn allow for faster public health responses and targeted interventions.

The data presented demonstrate that HRM assays simultaneously targeting multiple mutations in a multiplex format specifically and sensitively discriminate different VOCs. In our analysis, for all primer sets, except that for S_del.156–157, sensitivity and specificity values were calculated using sequencing data as the reference standard and samples were excluded if sequence coverage did not include the relevant nucleotides. However, due to a lack of sequencing coverage over the site of mutation S_del.156–157 due to mismatches with the ARTIC V3 primer set, sensitivity and specificity values were calculated based on the assumption that samples identified as Delta by the EPI2ME Desktop Agent v3.3.0, did contain the lineage-defining D3 mutation.

The HRM approach does have some limitations in comparison to sequencing-based surveillance. Firstly, the HRM assay does not produce the genomic information that is needed for phylogenetic analysis and outbreak tracking, which have been critical for genomic-based epidemiology throughout the COVID-19 pandemic^30,31^. Secondly, the HRM assay requires targeted primer design and as such relies on a priori knowledge of the variants’ genome sequence. Therefore, we propose that this rapid and less complex test is used in conjunction with some level of NGS, and that in lower-resourced settings deploying HRM assays may create access to sustainable variant surveillance and allow more focussed decisions on when to use NGS, reducing burden of workloads in sequencing hubs and overall reducing the costs of undertaking molecular epidemiological surveillance.

Whilst the HRM assay cannot characterise and define a new variant prior to identification by NGS, it can indicate samples with sequences that may be different from the currently prevalent variant in a geographical area. As shown, we used the HRM in a field trial when the Alpha VOC was emerging to replace 19A as the most prevalent variant circulating in the UK (January 2021 saw a week-on-week increase in the proportion of cases sequenced associated with Alpha VOC from 57% on 4 January 2021 to 87% by 1 February 2021). However, amid the emergence of the Alpha VOC, the HRM assay identified specimens containing SARS-CoV-2 sequences that were non-19A and non-Alpha VOC, which were resolved by NGS as the less common 20E(EU1) variant. 20E(EU1) initially expanded in Spain and spread across Europe in association with tourism travel^32^ but at the time of sample collection accounted for < 25% of cases in the UK (strain prevalence data from CoVariants.org). Therefore, whilst HRM does not identify new emerging variants, it can signal where samples may warrant further investigation by NGS, again allowing more targeted use of NGS in surveillance work.

Beyond the initial assay description presented here, as new variants arise, we aim to continually update the assay with new primer sets for the identification of new VOCs. By designing amplicons with Tm values that dovetail with existing assays it is possible to ensure they will work in multiplex with existing primers, maximising the functionality of the assay. Up-to-date primer sets, with their respective Tm values, potential multiplex partner primers, and assay conditions will be made freely available online at [link to be confirmed] as an open-access resource. The authors are also dedicated to assisting any laboratories looking to implement these tests as part of their SARS-CoV-2 surveillance or research.

## Data Availability

All sequence data is deposited in BioProject PRJNA936677 [https://www.ncbi.nlm.nih.gov/bioproject/PRJNA936677].
